# Overlapping genetic susceptibility variants between three autoimmune disorders: rheumatoid arthritis, type 1 diabetes and coeliac disease

**DOI:** 10.1186/ar3139

**Published:** 2010-09-20

**Authors:** Stephen Eyre, Anne Hinks, John Bowes, Edward Flynn, Paul Martin, Anthony G Wilson, Ann W Morgan, Paul Emery, Sophia Steer, Lynne J Hocking, David M Reid, Pille Harrison, Paul Wordsworth, Wendy Thomson, Jane Worthington, Anne Barton

**Affiliations:** 1 Arthritis Research UK-Epidemiology Unit, Stopford Building, Oxford Road, The University of Manchester, Manchester M13 9PT, UK; 2 School of Medicine & Biomedical Sciences, Sheffield University, Beech Hill Road, Sheffield S10 2JF, UK; 3 NIHR-Leeds Musculoskeletal Biomedical Research Unit, Leeds Institute of Molecular Medicine, University of Leeds, Beckett Street, Leeds LS2 9JT, UK; 4 Clinical and Academic Rheumatology, Kings College Hospital NHS Foundation Trust, Denmark Hill, London SE5 9RS, UK; 5 Musculoskeletal and Genetics Section, Division of Applied Medicine, University of Aberdeen, Foresterhill, Aberdeen AB25 2ZD, UK; 6 University of Oxford Institute of Musculoskeletal Sciences, Botnar Research Centre, Windmill Road, Oxford OX3 7LD, UK

## Abstract

**Introduction:**

Genome wide association studies, replicated by numerous well powered validation studies, have revealed a large number of loci likely to play a role in susceptibility to many multifactorial diseases. It is now well established that some of these loci are shared between diseases with similar aetiology. For example, a number of autoimmune diseases have been associated with variants in the *PTPN22, TNFAIP3 *and *CTLA4 *genes. Here we have attempted to define overlapping genetic variants between rheumatoid arthritis (RA), type 1 diabetes (T1D) and coeliac disease (CeD).

**Methods:**

We selected eight SNPs previously identified as being associated with CeD and six T1D-associated SNPs for validation in a sample of 3,962 RA patients and 3,531 controls. Genotyping was performed using the Sequenom MassArray platform and comparison of genotype and allele frequencies between cases and controls was undertaken. A trend test *P*-value < 0.004 was regarded as significant.

**Results:**

We found statistically significant evidence for association of the *TAGAP *locus with RA (*P *= 5.0 × 10^-4^). A marker at one other locus, *C1QTNF6*, previously associated with T1D, showed nominal association with RA in the current study but did not remain statistically significant at the corrected threshold.

**Conclusions:**

In exploring the overlap between T1D, CeD and RA, there is strong evidence that variation within the *TAGAP *gene is associated with all three autoimmune diseases. Interestingly a number of loci appear to be specific to one of the three diseases currently studied suggesting that they may play a role in determining the particular autoimmune phenotype at presentation.

## Introduction

Rheumatoid arthritis (RA) is an auto-immune, inflammatory joint condition, affecting 0.8% of the UK population [1]. It has long been established that the major genetic predisposition to this disease is contributed by variants of the class II HLA gene, *HLA DRB1*. Both type 1 diabetes (T1D) and coeliac disease (CeD) are also auto-immune, inflammatory diseases for which the major genetic contribution arises from the major histocompatibility complex and which are also characterised by autoantibody formation [2].

Recent genome wide association and subsequent replication studies in these three diseases have revealed a large number of well validated, non-HLA genetic risk loci providing an opportunity to explore the possibility of overlapping susceptibility between them [3]. Testing confirmed T1D susceptibility loci in CeD samples has already resulted in the identification of novel CeD loci [2]. A previous study exploring the overlap of CeD and RA susceptibility loci identified association to six regions [4]. We have recently reported our findings of the testing of putative T1D susceptibility loci in RA, which identified *AFF3 *as a novel RA susceptibility locus [5]. However, exploring the overlap between the three diseases in more detail may provide a further opportunity to elucidate the genetic similarities, and differences, between these common autoimmune diseases.

We, therefore, tested 14 validated susceptibility loci from CeD and T1D in a large, well powered cohort of British Caucasian patients with RA and independent controls to explore the overlap between loci identified in all three conditions.

## Materials and methods

### Samples

Clinical characteristics of the RA case-control cohort tested have been described previously [6]. RA cases satisfied American College of Rheumatology (ACR) classification criteria for RA modified for genetic studies, all provided informed consent and were collected with ethical committee approval (North-West Multi-Centre Research Ethics Committee (MREC 99/8/84) and the University of Manchester Committee on the Ethics of Research on Human Beings) [7,8]. Their clinical characteristics are shown in Table S1 in Additional file 1.

### SNP selection

SNP markers with evidence for association with CeD susceptibility (*P *< 1 × 10^-5^) in reference [2] were selected for genotyping in a cohort of RA and control samples. Where more than one SNP was associated at a particular locus with CeD, the most associated variant was selected for genotyping in the RA case-control cohort.

SNP markers with evidence for association in T1D in the same reference, but not genotyped as part of our previous work, were also selected for genotyping in the current cohort of RA cases and controls.

### Genotyping

SNP markers were genotyped using the iPlex chemistry on the Sequenom platform according to the manufacturer's instructions (Sequenom, San Diego, California, USA) [9]. Duplicate samples and negative controls were included on the plates to ensure genotyping accuracy. Additional quality control measures were introduced such that only SNP markers and samples exceeding a 90% genotyping success threshold were included in any subsequent analysis.

### Analysis

The power to detect association in the current cohort assuming the same odds ratio (OR) as was reported in the CeD disease study was calculated using Quanto [10]. Departure from Hardy-Weinberg equilibrium was tested for all SNP markers. Genotype frequencies were compared between RA cases and controls using the trend test implemented in STATA (StataCorp LP, Texas, USA). Conditional logistic regression was used to assess whether effects were independent where two SNPs had been tested at the same locus.

## Results

### SNP selection

Eight CeD SNPs were selected for genotyping as they had not previously been examined for association with RA (Table 1). We have previously reported our results for most but not all of the T1D loci tested in a recent study exploring the genetic overlap between CeD and T1D [5]. For completeness, six T1D associated SNPs not previously investigated for association with RA in the current data set were, therefore, also tested (Table 2).

**Table 1:** Results of association testing of previously reported CeD-associated loci with RA susceptibility

SNP	CHR	position	Gene	MAF cases	MAF controls	P_HWE	case_11	case_12	case_22	control_11	control_12	control_22	P_trend	Allelic OR (95% CI)
rs2816316	1	190803436	*RGS1*	0.17	0.18	0.31	119 (3.3)	1027 (28.2)	2501 (68.6)	99 (3.4)	835 (28.5)	1993 (68.1)	0.65	0.98 (0.89 to 1.07)
rs917997	2	102437000	*IL18RAP*	0.23	0.22	0.87	208 (5.7)	1,285 (35.2)	2,154 (59.1)	147 (5.0)	1,013 (34.6)	1,771 (60.4)	0.17	1.06 (0.98 to 1.15)
rs6441961	3	46327388	*CCR3*	0.31	0.31	0.76	350 (9.6)	1,582 (43.3)	1,720 (47.1)	280 (9.6)	1,239 (42.3)	1,409 (48.1)	0.52	1.03 (0.95 to 1.1)
rs333	3	46389951	*CCR5*	0.1	0.11	0.38	23 (0.7)	689 (19.6)	2,802 (79.7)	35 (1.1)	642 (20.6)	2,444 (78.3)	0.08	0.91 (0.81 to 1.01)
rs17810546	3	161147744	*IL12A*	0.12	0.12	0.66	52 (1.4)	736 (20.2)	2,862 (78.4)	45 (1.5)	616 (21.1)	2,264 (77.4)	0.32	0.95 (0.85 to 1.05)
rs1464510	3	189595248	*LPP*	0.45	0.44	0.72	697 (19.9)	1,730 (49.4)	1,077 (30.7)	617 (19.9)	1,524 (49.1)	966 (31.1)	0.82	1.01 (0.94 to 1.08)
rs182429	6	159389562	*TAGAP*	0.42	0.45	0.82	600 (17.0)	1,770 (50.2)	1,159 (32.8)	590 (20.5)	1,421 (49.3)	870 (30.2)	0.0005	0.88 (0.82 to 0.95)
rs653178#	12	110492139	*SH2B3*	0.49	0.51	0.68	914 (25.1)	1,744 (47.9)	985 (27.0)	771 (26.4)	1,449 (49.6)	703 (24.1)	0.02	0.92 (0.86 to 0.98)

# r^2 ^= 1 with rs3184504; 11 = homozygote for minor allele, 12 = heterozygote, 22 = homozygote for major allele; CHR, chromosome; MAF, minor allele frequency; P_HWE, *P*-value for Hardy Weinberg equilibrium test

**Table 2:** Association testing of six T1D associated SNPs not previously investigated for association with RA in the current cohort

SNP	CHR	position	Gene	MAF cases	MAF controls	P_HWE	case_11	case_12	case_22	control_11	control_12	control_22	P_trend	Allelic OR (95% CI)
rs11755527	6	91014952	*BACH2*	0.46	0.46	0.91	729 (20.8)	1,790 (51.0)	990 (28.2)	669 (21.5)	1,551 (49.9)	891 (28.6)	0.86	0.99 (0.93 to 1.06)
rs689	11	2138800	*INS*	0.28	0.28	0.54	287 (8.2)	1,411 (40.1)	1,818 (51.7)	254 (8.2)	1,246 (40.0)	1,612 (51.8)	0.95	1.00 (0.93 to 1.08)
rs12708716	16	11087374	*CLEC16A*	0.36	0.36	0.63	507 (12.9)	1,791 (45.4)	1,643 (41.7)	453 (12.9)	1,596 (45.5)	1,455 (41.5)	0.88	1.00 (0.93 to 1.06)
rs3825932	15	77022501	*CTSH*	0.32	0.32	0.77	368 (10.5)	1,506 (43.1)	1,619 (46.3)	323 (10.4)	1,366 (44.1)	1,407 (45.4)	0.62	0.98 (0.91 to 1.06)
rs3788013	21	42714397	*UBASH3A*	0.45	0.43	0.86	705 (20.1)	1,752 (49.9)	1,052 (30.0)	590 (19.0)	1,522 (49.0)	996 (32.0)	0.07	1.07 (1.00 to 1.14)
rs229541	22	35921264	*C1QTNF6*	0.45	0.43	0.49	726 (20.6)	1,693 (48.1)	1,100 (31.3)	564 (18.1)	1,548 (49.6)	1,007 (32.3)	0.04	1.08 (1.00 to 1.15)

11 = homozygote for minor allele, 12 = heterozygote, 22 = homozygote for major allele; CHR, chromosome; MAF, minor allele frequency; P_HWE, *P*-value for Hardy Weinberg equilibrium test

### Genotyping

Genotyping was undertaken in an available validation cohort of 3,962 RA cases and 3,531 controls from the UK. A *P*-value of 0.004 was selected as the nominal significance threshold. The study had between 67% and 99% power to detect association with the CeD loci in this sample at the *P *< 0.004 threshold assuming the same allele frequencies and effect sizes as reported with CeD (Table S2 in Additional file 1). Duplicate samples showed >99% concordance. All samples and SNPs met the quality control threshold of >90% genotyping success. All SNP genotype frequencies conformed to Hardy-Weinberg expectations in the controls.

One marker with previous evidence for association with CeD was also associated with RA in the current cohort: rs182429 mapping to the *TAGAP *gene OR 0.88, 95% confidence intervals (CI) 0.82 to 0.95; *P*-trend 0.0005) (Table 1). The *TAGAP *locus has previously been identified as an RA susceptibility locus following meta-analysis of genome-wide association studies in RA followed by validation in independent samples, which included samples from the current study [11]. However, a different SNP marker (rs394581) was tested in that study. The two SNPs were in moderate linkage disequilibrium (LD) (r^2 ^= 0.32; D' = 0.73) in the control samples tested here but conditional logistic regression analysis suggested that the association arises primarily from the CeD SNP, rs182429 (*P-*value for rs394581 after conditioning on rs182429 = 0.90).

Of the T1D SNPs tested, only rs229541, mapping to *C1QTNF6*, showed nominal evidence for association but this did not retain statistical significance at the threshold selected (OR 1.08, 95% CI 1.0 to 1.15, *P *= 0.04) (Table 2). Interestingly, this gene lies close to the *IL2RB *gene, which has previously been associated with RA susceptibility [6]. The apparent lack of association detected with T1D susceptibility loci in our sample may result from limited power to detect the effect sizes reported with the larger T1D samples: for three of the six loci, there was <40% power to detect association at the *P *< 0.004 threshold (Additional file 1 Table S2). However, association with the *INS, CLEC16A *and *CTSH *loci can be excluded with more confidence.

The three autoimmune diseases examined in the current study are characterised by the presence of autoantibodies and, interestingly, the association of the *TAGAP *variant is stronger in the subgroup of RA patients with anti-cyclic citrullinated peptide (anti-CCP) antibodies (*P*-trend = 0.0001, allelic OR 0.83 95% CI 0.75 to 0.91), suggesting a possible role in control of B cell function.

## Discussion

By investigating the genetic overlap of CeD genes with RA, we have identified the *TAGAP *locus as being associated with RA. Our findings confirm and refine the results of a previous study in RA that also showed association to the *TAGAP *region but with a different variant; our results show that the same SNP that is associated with CeD and T1D also shows stronger evidence for association with RA [11]. Data from the current study extend that of previous work identifying that *CTLA-4*, the *IL2_21 *region, 6q23 (*TNFAIP3*), *SH2B3*, *PRKCQ*, *MMEL1 *and now *TAGAP *are susceptibility loci that are common to the three autoimmune diseases examined in the current study: T1D, CeD and RA [2,5].

The current study is limited by the fact that, because the identification of novel susceptibility markers is progressing rapidly, a number of additional CeD and T1D susceptibility loci have been identified more recently, which have not been tested as yet for association with RA [12-14]. Hence, this is not a comprehensive examination of the overlap among all T1D, CeD and RA loci. However, of the loci tested so far, there appears to be greater overlap between T1D/CeD and T1D/RA than between CeD and RA. This may reflect the fact that larger sample sizes were used to investigate T1D (> 8,000 T1D cases vs >9,000 controls) than CeD (2,500 cases vs >9,000 controls), thereby reducing the possibility of false negative results in the T1D series. The lack of association of some of the CeD loci with RA could reflect lack of power, particularly if *winner's curse *has resulted in an overestimation of the effect sizes originally reported in the CeD studies. However, effect sizes observed when data from four GWAS of CeD were combined by meta-analysis were similar to those reported in the study by Smyth *et al*., which was used to select the CeD variants for testing in the current study [2,13]. That suggests that *winner's curse *and over-estimation of effects sizes in CeD cannot explain the lack of association observed with RA.

The *TAGAP *minor allele confers protection against RA, similar to previous reports of T1D but contrasting with CeD in which the minor allele is associated with risk. Our results confirm and extend the evidence that there are common *autoimmune susceptibility *genes but suggest that the overlap of RA is stronger with TID than CeD.

Relatively little is known about the *TAGAP *gene, which encodes a protein (T-cell activation RhoGTPase activating protein) transiently expressed in activated T cells, suggesting that it may have a role in immune regulation [2].

The *TAGAP *gene has previously been associated with RA following meta-analysis of US and European RA cohorts, although a different variant mapping to the same locus was genotyped [11]. Nonetheless, the same UK samples tested as part of the current study were also included in the validation phase of the meta-analysis and so data for both variants was available. The results of conditional logistic regression analysis suggest that the primary effect is with the CeD variant tested in the current study and that the association reported previously with rs394581, may have arisen due to LD, at least in the UK samples.

**Figure 1:**
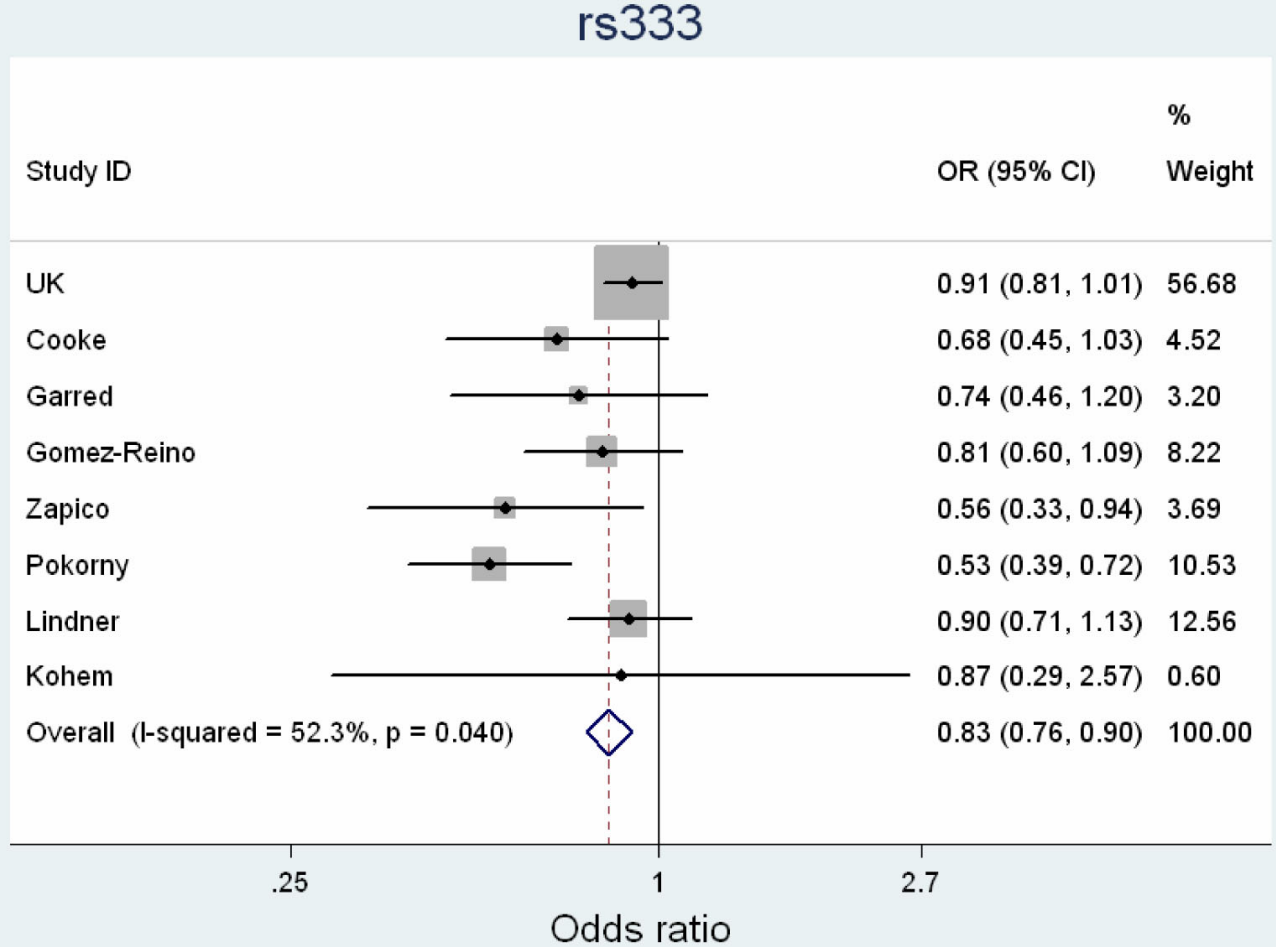
**Meta-analysis of studies investigating a deletion in the *CCR5 *gene with RA susceptibility**.

It is interesting to note that the SNP tested at the *CCR5 *locus which is associated with both T1D and CeD, also showed a trend towards association with RA. A previous meta-analysis of studies in RA investigating this deletion variant within the gene is suggestive of association with RA [15]. A combined meta-analysis, including data from the previous meta-analysis, two subsequent reports [16] and the current data, reveals statistically significant evidence for association (*P *= 1.4 × 10^-5^) but because of heterogeneity between the studies, these results should be interpreted with caution (Figure 1).

Exploring the genetic overlap between related diseases may reveal key common pathways that could suggest therapeutic targets applicable to more than one disease. However, the susceptibility loci unique to a particular disease are also of interest; differences may reflect genuine specificity between the diseases and may influence what determines the particular autoimmune phenotype. Of the confirmed non-HLA RA susceptibility loci (reviewed in [17]), only the *CD40 *and *TRAF1/C5 *variants have not been reported to be associated with either CeD or T1D although *CD40 *is associated with autoimmune thyroiditis [18]. Even the *TRAF1/C5 *association is not unique to RA as there have been reports of association with juvenile idiopathic arthritis and systemic lupus erythematosus [19,20]. Despite the inherent bias arising from the fact that loci associated with one autoimmune disease are often subsequently targeted for investigation in other autoimmune diseases, the degree of genetic overlap is remarkable. One possible explanation may be that specificity of the autoimmune phenotype is determined by the particular HLA associations of each disease that could either directly or indirectly influence the response to environmental agents. This hypothesis would require further investigation in well-powered cohorts with both genetic and reliable environmental data.

In summary, we report association of RA with the *TAGAP *gene, identified through targeting loci previously associated with CeD. The findings identify that the same SNP associated with T1D and CeD shows stronger association with RA than a previously reported variant and extend the evidence for overlap between *autoimmunity genes*.

## Conclusions

The *TAGAP *gene, previously associated with both T1D and CeD, is also associated with RA susceptibility. This confirms the finding of a previous study showing association of the *TAGAP *locus with RA but suggests that the variant associated with autoimmune diseases is more strongly associated than that reported previously.

## Abbreviations

AFF3: AF4/FMR2 family member 3; C1QTNF6: C1q and tumour necrosis factor related protein 6; CCR5: cytokine-chemokine receptor 5; CD40: CD40 molecule; CED: coeliac disease; CHR: chromosome; CI: confidence interval; CLEC16A: C-type lectin domain family 16; CTLA4: cytotoxic T lymphocyte activated 4; CTSH: cathepsin H; HLA: human leucocyte antigen; HWE: Hardy Weinberg equilibrium; IL: interleukin; IL2RB: interleukin 2 receptor beta; INS: insulin;LD: linkage disequilibrium; MAF: minor allele frequency; OR: odds ratio; PTPN22: protein tyrosine phosphatise non-receptor 22; RA: rheumatoid arthritis; SNP: single nucleotide polymorphism; T1D: type 1 diabetes; TAGAP: T-cell activation RhoGTPase activating protein; TNFAIP3: tumour necrosis factor activation induced protein 3; TRAF1/C5: tumour necrosis receptor activation factor 5/Complement 5; WTCCC: Wellcome Trust Case Control Consortium.

## Competing interests

The authors declare that they have no competing interests.

## Authors' contributions

SE, WT, JW and AB conceived the study. EF and PM performed the genotyping, while AH and JB undertook the statistical analysis. YEAR and BIRAC consortia, AGW, AWM, PE, SS, LJH, DMR, PH, PW, JW and AB provided samples. SE and AB drafted the manuscript, and all authors contributed to and approved the final version.

## Supplementary Material

Additional file 1 **Overlapping genetic susceptibility variants between three autoimmune disorders - supplementary information**. The file contains tables showing the clinical characteristics of the RA patient samples tested as well as the power of the study for each SNP tested. Click here for file
