# Supplementary material for: Overlapping genetic susceptibility variants between three autoimmune disorders: rheumatoid arthritis, type 1 diabetes and coeliac disease
Source: Arthritis Res Ther. 2010 Sep 20;12(5):R175. doi: 10.1186/ar3139 (PMC2991006; doi:10.1186/ar3139)
Supplement: Additional file 1 — Overlapping genetic susceptibility variants between three autoimmune disorders - supplementary information. The file contains tables showing the clinical characteristics of the RA patient samples tested as well as the power of the study for each SNP tested. [file ar3139-S1.DOC]

Overlapping genetic susceptibility variants between three autoimmune disorders: rheumatoid arthritis, type 1 diabetes and coeliac disease.

Stephen Eyre*^1^, Anne Hinks^1^, John Bowes^1^, Edward Flynn^1^, Paul Martin^1^, YEAR Consortium^7^, BIRAC Consortium^8^, Anthony G Wilson^2^, Ann W Morgan^3^, Paul Emery^3^, Sophia Steer^4^, Lynne J Hocking^5^, David M Reid^5^, Pille Harrison^6^, Paul Wordsworth^6^, Wendy Thomson ^1^, Jane Worthington^1^, Anne Barton^1^.

^7^YEAR Consortium:

**Management Team:** Professor Paul Emery, Professor Philip Conaghan, Dr Mark Quinn, Dr Ann Morgan, Anne-Maree Keenan, Dr Elizabeth Hensor, Julie Kitcheman

**Consultants:** Dr Andrew Gough, Dr Michael Green, Dr Richard Reece, Dr Lesley Hordon, Dr Philip Helliwell, Dr Richard Melsom, Dr Sheelagh Doherty, Dr Ade Adebajo, Dr Andrew Harvey, Dr Steve Jarrett, Dr Gareth Huson, Dr Amanda Isdale

Dr Mike Martin, Dr Zinaid Karim, Dr Dennis McGonagle, Dr Colin Pease, Dr Sally Cox

**SpRs:** Dr Victoria Bejarano, Dr Jackie Nam

**Nurses:** Claire Brown, Christine Thomas, David Pickles, Alison Hammond, Beverley Neville, Alan Fairclough, Caroline Nunns, Anne Gill, Julie Green, Belinda Rhys-Evans, Barbara Padwell, Julie Madden, Lynda Taylor, Sally Smith, Heather King, Jill Firth, Jayne Heard, Linda Sigsworth

**Lab Staff:** Diane Corscadden, Karen Henshaw, Lubna-Haroon Rashid, Stephen G Martin, James I Robinson

^8^Biologics in RA Control (BIRAC) consortium members are:

**Derbyshire Royal Infirmary, Derby** (Dr. LJ Badcock, Dr. CM Deighton, Dr. SC O'Reilly, Dr. MR Regan, Dr. Snaith, Dr.GD Summers, Dr. RA Williams)

**Russells Hall Hospital, Dudley** (Dr. J. Delamere, Dr. K. Douglas, Dr. N. Erb, Prof. G.D. Kitas, Dr. R. Klocke, Dr. A. Pace, Dr. A. Whallett.)

**Glasgow Royal Infirmary and Gartnavel Hospital**, **Glasgow** (Dr D Porter, Dr J Hunter, Dr MM Gordon, Dr M Gupta, Prof H Capell, Prof R Sturrock, Prof I McInnes, Dr R Madhok, Dr M Field)

**Hope Hospital, Salford** (Dr. R Cooper, Dr A Herrick, Dr T O’Neil, Prof A Jones, Dr. R. Benitha)

**East Cheshire NHS Trust, Macclesfield** (Dr A Barton, Dr S Knight, Prof D Symmons)

**Manchester Royal Infirmary, Manchester** (Dr RM Bernstein, Dr I N Bruce, Dr K Hyrich, Prof A Silman)

**Norfolk & Norwich University Hospital, Norfolk** (Dr. K Gaffney, Prof. AJ Macgregor, Dr. T. Marshall, Dr. P Merry, Prof. DGI Scott)

**Poole General Hospital, Poole** (Dr PW Thompson, Dr SC Richards)

**Queen Alexandra Hospital, Portsmouth** (Dr. RG Hull, Dr. JM Ledingham, Dr. F Mccrae, Dr. MR Shaban, Dr. AL Thomas, Dr S Young Min)

**St Helens Hospital, St Helens** (Dr. V E Abernethy, Dr. J K Dawson, Dr. M Lynch)

**Haywood Hospital, Stoke-On-Trent** (Dr EH Carpenter, Dr. PT Dawes, Dr. C Dowson, Dr. A Hassell, Prof. EM Hay, Dr. S Kamath, Dr. J Packham, Dr. E. Roddy, Dr. MF Shadforth)

Supplementary Table 1. Clinical characteristics, where available, of the RA subjects tested in the validation study, by centre of recruitment.

| **Cohort** | **Total**  **N** | **Manchester**  **N** | **Aberdeen**  **N** | **Leeds**  **N** | **Sheffield**  **N** | **London**  **N** | **Oxford**  **N** |
| --- | --- | --- | --- | --- | --- | --- | --- |
| Controls | 3531 | 832 | 825 | 422 | 929 | - | 523 |
| Cases | 3962 | 1051 | 131 | 844 | 927 | 276 | 733 |
| Age at onset | 48.8±16.0 | 47.6±17.2 | 47.9±12.2 | 58.9±14.0 | 43.9±14.9 | 47.6±13.9 | 48.2±14.4 |
| **Case**  **Characteristic** | **Total**  **N (%)** | **Manchester**  **N (%)** | **Aberdeen**  **N (%)** | **Leeds**  **N (%)** | **Sheffield**  **N (%)** | **London**  **N (%)** | **Oxford**  **N (%)** |
| Gender  M  F | 1106 (28.2)  2822 (71.8) | 286 (27.2)  765 (72.8) | 40 (30.5)  91 (69.5) | 270 (32.0)  574 (68.0) | 248 (27.5)  654 (72.5) | 55 (20.6)  212 (79.4) | 207 (28.2)  526 (71.8) |
| RF  Negative  Positive | 1039 (27.9)  2684 (72.1) | 280 (26.9)  762 (73.1) | 30 (24.0)  95 (76.0) | 243 (33.6)  480 (66.4) | 226 (27.1)  607 (72.9) | 96 (35.9)  171 (64.1) | 164 (22.4)  569 (77.6) |
| Anti-CCP  Negative  Positive | 731 (32.0)  1550 (67.9) | 364 (37.8)  599 (62.2) | -  - | 167 (38.7)  265 (61.3) | 200 (22.6)  686 (77.4) | -  - | -  - |
| SE (no. copies)  0  1  2 | 766 (25.1)  1506 (49.5)  773 (25.4) | 256 (25.6)  521 (52.0)  224 (22.4) | -  -  - | 96 (27.1)  184 (52.0)  74 (20.9) | 186 (21.2)  408 (46.5)  283 (32.3) | 58 (22.0)  129 (48.9)  77 (29.1) | 170 (31.0)  264 (48.1)  115 (20.9) |

**Supplementary Table 2. Power of the current study to detect same effect size as reported previously**

|  |  |  |  |  |  |  |  |
| --- | --- | --- | --- | --- | --- | --- | --- |
|  | rs id | disease | locus | Allele Frq. | effect size (OR) | Power (RA study) |  |
|  | rs2816316 | CD | RGS1 | 0.182 | 0.72 | 99.99 |  |
|  | rs917997 | CD | IL18RAP | 0.221 | 1.29 | 99.56 |  |
|  | rs6441961 | CD | CCR3 | 0.301 | 1.21 | 90.27 |  |
|  | rs17810546 | CD | IL12A | 0.123 | 1.35 | 99.78 |  |
|  | rs1464510 | CD | LPP | 0.456 | 1.23 | 88.23 |  |
|  | rs6822844 | CD | IL2_21 | 0.176 | 0.71 | 99.99 |  |
|  | rs1738074 | CD | TAGAP | 0.437 | 1.21 | 83.03 |  |
|  | rs3184504 | CD | SH2B3 | 0.484 | 1.21 | 77.37 |  |
|  | rs3087243 | CD | CTLA-4 | 0.452 | 0.85 | 67.66 |  |
|  | rs333 | CD | CCR5 | 0.119 | 0.79 | 90.30 |  |
|  | rs11755527 | T1D | BACH2 | 0.465 | 1.13 | 32.58 |  |
|  | rs689 | T1D | INS | 0.293 | 0.42 | 99.99 |  |
|  | rs3825932 | T1D | CTSH | 0.318 | 0.86 | 67.27 |  |
|  | rs12708716 | T1D | CLEC16A | 0.351 | 0.81 | 95.68 |  |
|  | rs3788013 | T1D | UBASH3A | 0.433 | 1.13 | 35.63 |  |
|  | rs229541 | T1D | CTQTNF6 | 0.428 | 1.12 | 29.71 |  |
|  |  |  |  |  |  |  |  |
|  | Power estimated based on 3962 cases and 3531 controls (ratio=0.89). | | | | |  |  |
|  | Control MAF was used for calculations | | |  |  |  |  |
|  | Prevalence = 0.01 | | | | | |  |
|  | Dominant Model | | | | | |  |
|  | alpha = 0.004 |  |  |  |  |  |  |
|  |  |  |  |  |  |  |  |
|  | All estimates performed using QUANTO | | |  |  |  |  |

CD – Coeliac disease; T1D = type 1 diabetes
